# Estimation and consumption pattern of free sugar intake in 3-year-old Irish preschool children

**DOI:** 10.1007/s00394-019-02056-8

**Published:** 2019-07-19

**Authors:** Michael Crowe, Michael O’Sullivan, Oscar Cassetti, Aifric O’Sullivan

**Affiliations:** 1 Division of Restorative Dentistry and Periodontology, Dublin Dental University Hospital, Trinity College Dublin, Lincoln Place, Dublin 2, Ireland; 2 UCD Institute of Food and Health, 2.05 Science Centre, South, UCD, Belfield, Dublin, Ireland

**Keywords:** Free sugars, Dietary survey, WHO, Preschoolers

## Abstract

**Purposes:**

Dietary free sugars (FS) are the most important risk factor for dental caries and can contribute to excess energy intake. Measuring FS intake is limited by food composition databases and appropriate dietary assessment methods. The aim of this analysis was to estimate total sugar (TS) and FS intakes for Irish pre-schoolers and examine the proportion of dietary TS and FS captured using a short food questionnaire (SFQ).

**Methods:**

This is a secondary analysis of 3-year-old children from two national surveys; Growing Up in Ireland (GUI), *N* = 9793 of whom 49% were girls and the National Preschool Nutrition Survey (NPNS), *N* = 126 and 52% were girls. GUI used SFQs and NPNS used semi-weighed food diaries to collect dietary data from 3-year-old children. Dietary intake databases were linked using an established approach. Mean daily TS and FS intakes and frequency were calculated, and consumption patterns from foods and meals are presented. The proportion of foods that were *covered* or *non*-*covered* by the GUI SFQ was calculated by comparison with the NPNS food diary.

**Results:**

75% of 3 year-olds had FS intake greater than the maximum recommended by WHO guidelines for free sugar intake, while 4% met the lower threshold. The median frequency of TS and FS consumption was 5.0 (4.0–6.0) and 4.0 (3.0–5.0) times/day. Less than one-quarter of TS intake (g/day) was *non*-*covered* by the GUI SFQ while less than one-third of FS intake was *non*-*covered.*

**Conclusions:**

A large majority of 3-year-old Irish children do not meet the WHO recommended guidelines for FS intake and almost none meet the desired conditional recommendation. SFQs only capture two-thirds of FS intake at this early age.

## Introduction

High intakes of dietary sugars have been implicated as a public health issue with concerns regarding their contribution to increased obesity prevalence and negative impact on oral health [1–3]. Dietary free sugars (FS) are the most important risk factor in the development of dental caries [4, 5] and can contribute to excess energy intake with little nutrient benefit. In 2015 the WHO updated their recommendations to include a reduction in FS intake throughout the life course to < 10% of Total Energy Intake (TEI) (strong recommendation), noting a further reduction to < 5% of TEI would provide additional health benefits (conditional recommendation) [6].

The term “sugar” refers to sucrose or “table sugar” while total sugars (TS) can be defined as the sum of natural and added sugars (AS) in a food or beverage [7]. Intrinsic sugar is a term which refers to sugars that are incorporated in the structure of intact fruit and vegetables [7]. AS include those sugars added during the production or processing of food and not naturally found in the food product [8] and is the term defined by the Food and Drug Administration (FDA) in the USA. Free sugars (FS), which is the preferred term used by the WHO, includes sugars naturally present in honey, syrups, fruit juices and fruit juice concentrates as well as AS. Scientific Advisory Committee on Nutrition advises that the average population intake of FS should not be greater than 5% of TEI from 2 years upwards and that for children there should be minimal consumption of sugar-sweetened drinks [2].

Worldwide, there are very few food databases that contain information regarding AS or FS levels [8–11]. In most national studies, a large proportion of children exceeded the recommended guidelines for AS/FS consumption [11–17]. Consequently, some professional paediatric bodies have made practical recommendations for reducing AS/FS intake in children [18].

The early introduction and high consumption of cariogenic (dental caries or tooth decay causing) food and drink have been observed since the 1970s [19, 20] when, in most European countries, sugar intake per capita reached a peak [21]. As well as concerns about establishing unhealthy eating patterns at an early age that may influence weight status [22], sugar intake is a key risk factor in the progression and reversal of early dental caries [23, 24]. While reducing the frequency of consumption of FS can assist in lowering dental caries risk, it is also necessary to reduce the amount of FS to reduce the risk of other non-communicable diseases related to excess sugar intake [4, 6, 25].

Given the difficulties and costs involved in collecting accurate dietary records, large nationally representative cohort surveys increasingly use limited dietary assessment instruments such as short food questionnaires (SFQ) to determine the intake of “healthy” and “unhealthy” foods. We have previously described a method that can be used to link matched datasets from two studies of 3 year-olds; Growing Up in Ireland (GUI) which estimated food intake of “healthy” and “unhealthy” foods using a SFQ and the National Preschool Nutrition Survey (NPNS) which collected food consumption data using a 4-day weighed food diary [26]. We reported food consumption estimated by the SFQ in GUI relative to the more detailed dietary assessment in NPNS. Using the linked datasets this paper aims to: (1) quantify FS intake in Irish preschool children; (2) determine the distribution of TS and FS consumption patterns (amount and frequency) compared to the WHO guidelines; (3) compare how well the SFQ in the GUI study captured the sources of TS/FS compared to NPNS and (4) identify the key food sources of TS/FS consumed as part of a main meal or snack.

## Methods

### Data source and participants

GUI, the largest children’s study ever undertaken in Ireland, is a nationally representative longitudinal survey that collected data, from an infant cohort (at 9 months of age) and a child cohort (at 9 years of age). The data used in this analysis was from the second wave of the infant cohort (at 3 years of age) which was collected between December 2010 and July 2011. The GUI study selected a random sample on a systematic basis, pre-stratified by marital status, county of residence, nationality and number of children from the National Child Benefits Register, which is a universal welfare entitlement in the Republic of Ireland [27]. The total sample consisted of 9793 cases, of whom 49% were girls. The primary caregiver was interviewed in the family home using Computer-Assisted Personal Interview questionnaires after written informed consent was obtained. Full details of the sample population, sample design, participant response, fieldwork/implementation, survey instruments and interviewer training are available at http://www.esri.ie/growing-up-in-ireland/ [27, 28].

National Preschool Nutrition Survey used a quota sampling approach to obtain a nationally representative sample of children within each of the four preschool age groups between 1 and 4 years of age [29]. The NPNS had a total sample of 500 children aged 2–4 years. Only the 3-year-old were included for this analysis and the final total number of participants was 126, of whom 52% were girls. In the NPNS study, a nutrition researcher visited the participant’s home on three occasions during the 4-day food record period. The methods employed for the NPNS study are available at http://www.iuna.net/, including details of the quality procedures that were used to help consistency and minimise error throughout the collection and manipulation of the food intake data [30].

Both studies were conducted according to guidelines laid down in the Declaration of Helsinki. Ethical approval for the GUI project was received from a Research Ethics Committee convened by the Department of Health and Children while approval for the Irish University Nutrition Alliance (IUNA)-NPNS project was obtained from the Clinical Research Ethics Committee of the Cork Teaching Hospitals, University College Cork. Data from GUI was obtained from the Irish Social Science Data Archive (ISSDA, University College Dublin) and the NPNS datafiles were obtained from the IUNA Database Management Committee.

### Data manipulation and analysis

The initial stages of data mapping, linkage and estimation of *covered* and *non*-*covered* GUI and NPNS food data were previously reported [26]. Initially, all GUI food groups were filled with information from the NPNS food datafile, all food categories in NPNS were sorted and filtered and consolidated into a single augmented food database. This was used as an input to execute a unidirectional mapping protocol. The new database allowed a more comprehensive estimation of the food items consumed by the GUI cohort. Full details of the data mapping protocols and all subsequent analyses are also available at Trinity College Dublin’s access to research archive [31].

A consumption was defined as any eating occasion of a food or drink (snack or main meal) and any entry in the food diary in NPNS was considered a consumption. The description *non*-*covered* indicated a food consumption in the more detailed NPNS that could not be mapped using a GUI food group, i.e., the food in NPNS was not matched by the same food in GUI. If there was a matching GUI food group that the food consumption could be mapped to in GUI the term *covered* was used i.e., the food in NPNS was matched by the same food group in GUI. Manual mapping of all the food groups in GUI with those from NPNS was carried out using a combination of direct food name/food description matching, fuzzy matching, or word search using each word of the food names/food description [26]. Using this mapped data all food groups in NPNS and GUI were then examined to determine the proportion of foods that were *covered* or *non*-*covered* by GUI. The initial aggregation was carried out at the subject and survey day levels. The resulting mapped database allowed for a more accurate estimation of the amount and frequency of food and drink consumed by 3-year-old in the GUI cohort using the NPNS survey data.

In NPNS there were 1652 food codes which were categorised into 77 food groups and further re-categorised into 19 food groups. In GUI, there were 15 food groups. Food groups for both GUI and NPNS were re-categorised as follows to highlight the main FS food sources: ‘bread and cereals’, ‘ready-to-eat-breakfast-cereals’ (‘RTEBC’), ‘cakes and biscuits’, ‘dairy products’, ‘desserts and puddings’, ‘fruit and vegetables’, ‘fruit juice and smoothies’, ‘sugar and syrups’, ‘chocolate confectionary’, ‘non-chocolate confectionary’, ‘soft drinks (non-diet)’, ‘soft drinks (diet)’ and ‘other’. ‘Dairy products’ included all milk, yoghurt, cheese and ice-cream products. ‘Soft drinks (non-diet)’ included carbonated beverages, squashes, cordials and fruit juice drinks. ‘Breads and cereals’ included all rice, pasta, grains and cereal-based products except RTEBC. All other food categories were grouped into ‘other foods’ [31].

### Free sugar estimation

The mapped GUI database was used to carry out an estimation of the FS content of all food and drink. The FS content of foods from NPNS was estimated using a modified version of the method described by Louie et al. [32]. The methodology devised by Louie et al. used a stepwise protocol to estimate AS starting with objective estimation (six steps) and then using more subjective measures (four steps) if objective data were not available. The methodology used in this analysis specifically estimated FS. All foods or beverages were classified on the basis of analytical data and ingredients in food products. For example, for foods and beverages where there was no unsweetened equivalent variety but the analytical data for lactose was available then FS was calculated as TS minus lactose. As the modified analysis in this paper aimed to estimate FS those sugars naturally present in honey, syrups, fruit juices and fruit juice concentrates were included when assessing the individual foods or beverages. Using the method devised by Louie et al. fruit juices were classified as 0 g AS whereas this analysis assigned all the sugar content of fruit juices as FS. Full details of the protocol used in this FS estimation are available at Trinity College Dublin’s access to research archive [31].

The TS and lactose estimates from the NPNS database were imported into RStudio and then exported as a.csv file before estimating FS content based on the modified methodology. Two researchers estimated FS in the NPNS food database. The results were compared and any differences between the researcher’s estimations were highlighted. The final decision on the FS content was made by a third person, the senior nutritionist. The FS estimates were then imported into the mapped GUI database. These FS estimations were also compared to a previously reported estimation of FS using the same cohort of 3-year old [10] and the distributions compared using the Kolmogorov–Smirnov test (*p* < 0.01) which indicated no significant difference in the two distributions. All statistical analyses were carried out using R Studio [33]. Quantitative analysis and metrics were carried out as outlined below.

After the data were imported into RStudio they were aggregated across subject ID, day of the week and day of the survey. To compare how well the GUI-SFQ captured TS and FS intakes the following metrics were computed: (1) total number of times when a consumption (*non*-*covered* by GUI) occurred, (2) total food weight for *non*-*covered* consumptions, (3) TS and FS (weight) for a *non*-*covered* consumption, (4) TS and FS (weight) for a *covered* (by GUI) consumption, (5) total number of times when a *covered* consumption occurred, (5) TS and FS, (6) total number of consumptions and (7) total food weight.

Total sugar and free sugar were determined by multiplying the weight of food consumed daily, aggregated at the subject level, by the percentage of TS or FS. The mean daily intake of TS and FS (g/day), frequency of consumption (median, interquartile range) and as a percentage of TEI, were presented as summary statistics. The percentage of consumers in each food group were calculated. The probability of consuming a food or drink as a snack or main meal was estimated by using the total count of snacks or main meals over all 4 days of the survey. The daily intake of TS and FS *covered* and *non*-*covered* by GUI food groups by amount (g/day) and as a percentage of TEI was presented as bar graphs. Sugar intake (TS and FS) as a percentage of TEI was calculated using 0.017 MJ/g as a conversion factor [34]. The percentage of the sample population with a FS intake greater than the WHO recommendations [6] were determined.

Data reported were for average daily intake (mean, SD) across the full sample and the percentage of consumers of each food category were included. Under-reporters were identified in the dataset using an energy intake to basal metabolic rate (BMR) cut-off. Body weight and height for each subject were inputted to standard equations for predicting basal metabolic rate (BMR) [35]. Children with a minimum energy intake cut-point of 1.28 × BMR were identified as under-reporters (37%) [36]. Under-reporters were not removed from the data. The probability of consuming a food group as part of a main meal or snack was calculated using the total number of meal types (snacks and main meals) that were recorded during the 4-day NPNS survey.

## Results

The total number of 3-year-olds in the infant cohort of the GUI survey was 9793 of whom 5024 (51%) were male while the total number of 3 year-olds in NPNS was 126 of which 61 (48%) were male. The daily intake of TS and FS for 3-year-old in NPNS by amount (g/day) and as a percentage of TEI is shown in Table 1. The estimated mean daily intake of TS and FS were 75.8 (SD 29.3) and 40.0 (SD 23.5) g/day which contributed 26.9% (SD 5.9) and 14.1% (SD 5.81) of the TEI, respectively. Three-quarters of 3-year-old consumed more than 10% TEI as FS (WHO recommended maximum FS intake). The lower recommended threshold of 5% was achieved by less than 4% of the sample. The median frequency of TS and FS consumption (as a meal or snack) was 5.0 (4.0–6.0) and 4.0 (3.0–5.0) times per day. The median frequency of total meal consumption was 5.0 (4.0–6.0).

**Table 1:** Daily intake of total and free sugar for 3-year-old children by amount (g/day), frequency (as a meal or snack) and as a percentage of Total Energy Intake (TEI)

	Mean	SD	P25	P50	P75
Total sugars (g/day)	75.8	29.3	54.6	75.3	132.7
Total sugar (frequency)	5.2	1.2	4.0	5.0	6.0
Energy from total sugars (%TEI)	26.9	5.9	22.4	26.9	31.2
Free sugar (g/day)	40.0	23.5	21.3	36.7	81.5
Free sugar (frequency)	3.9	1.4	3.0	4.0	5.0
Energy from free sugar (%TEI)	14.1	5.81	10.0	13.5	65.5

*P25* 25th percentile, *P50* 50th percentile (median), *P75* 75th percentile

### Key sugar food sources

**Fig. 1:**
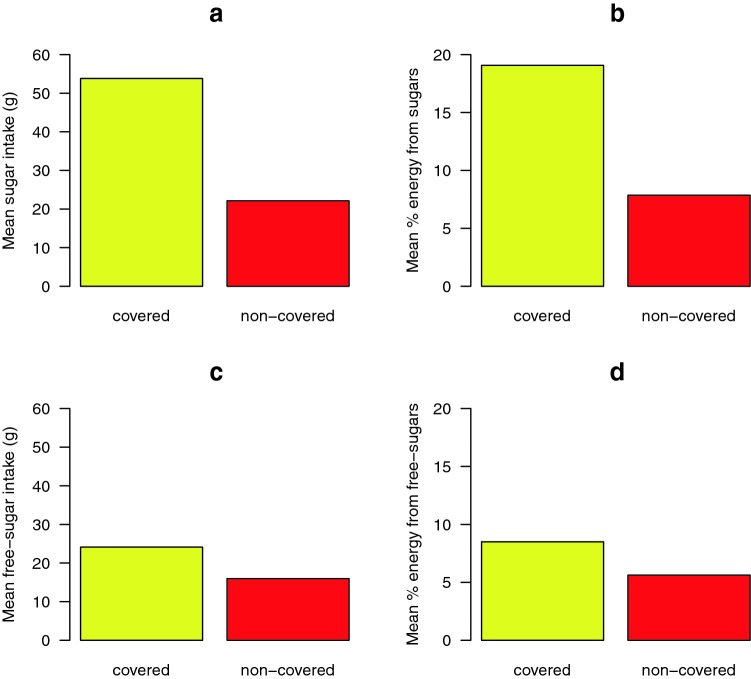
Daily intake of total and free sugar, *covered* and *non*-*covered,* by GUI food groups for 3-year-old children by amount (g/day) (**a**, **c**) and as a percentage of Total Energy Intake (%TEI) (**b**, **d**)

The key sugar-contributing food sources of TS and FS intake are displayed in Table 2. The most important contributors (mean g/day,  %TEI), to TS intake were ‘dairy products’ (22.0 g/day, 7.6% TEI), ‘fruit and vegetables’ (17.3 g/day, 6.3% TEI), ‘fruit juice and smoothies’ (8.7 g/day, 3.1% TEI) and ‘confectionary’ (chocolate and non-chocolate) (5.8 g/day, 2.0% TEI). The most important contributors to FS intake, were ‘fruit juice and smoothies’ (8.4 g/day, 3.0% TEI), ‘dairy products’ (8.2 g/day, 2.8% TEI), ‘confectionary’ (chocolate and non-chocolate) (5.3 g/day, 2.0% TEI), and ‘soft drinks’ (including squashes, cordials and fruit juice drinks) (4.8 g/day, 1.8% TEI). The percentage of consumers varied from 25% for ‘desserts and puddings’ to 100% for ‘dairy products’, ‘bread and cereals’ and ‘fruit and vegetables’. ‘Chocolate confectionary’ and ‘non-chocolate confectionary’ were consumed by 60% and 45% of the total NPNS sample, respectively. ‘Dairy products’ were consumed by all the sample population while ‘fruit juice and smoothies’ and ‘soft drinks’ (including squashes, cordials and fruit juice drinks) were consumed by over 70% of the sample population. ‘RTEBC’ were consumed by more 92% of children and contributed 7.8% of the total FS intake. The combination of all ‘cakes, biscuits and confectionary’ contributed 37.8% of the total FS intake. There was a high probability (2:1) of consuming ‘chocolate confectionary’, ‘cakes and biscuits’ and ‘non-chocolate confectionary’ as a snack while this probability was the opposite (1:2) for consumption of ‘fruit juices and smoothies’, ‘dairy products’ and ‘soft drinks (non-diet)’ as a snack. ‘RTEBC’ were nearly always likely to be consumed as part of a main meal. Figure 1a–d shows the mean daily intake of all food sources of TS and FS *covered* or *non*-*covered* by the SFQ used in the GUI survey as g/day and as  %TEI. Less than one-quarter of the mean TS intake (g/day) was *non*-*covered* by GUI (Fig. 1a) while less than one-third of the mean FS intake was *non*-*covered* (Fig. 1c). The proportions were similar when expressed as a percentage of TEI (Fig. 1 b, d). The most commonly consumed food categories rich in sugar that were *non*-*covered* by GUI included ‘RTEBC’, ‘fruit juices’ and ‘sugars and syrups’.

**Table 2:** Contribution of key sugar-contributing food sources to total sugar and free sugar intake in 3-year-old children as weight (g/day), as a percentage of Total Energy Intake (%TEI), by percentage consumers and by probability of consumption as part of a snack or main meal

	Fruit juices and smoothies	Dairy	Soft Drinks (Non diet)	Chocolate confectionery	Cakes and biscuits	Non-chocolate confectionery	Sugar and syrups	Desserts and puddings	RTEBC	Other	Bread cereals	Fruit and vegetables
Consumers (%)	73.0	100.0	71.4	59.5	89.7	45.2	56.3	25.4	92.1	100.0	100.0	100.0
Total sugars Mean (SD)	8.7 (8.7)	22.0 (11.1)	4.8 (8.8)	3.6 (4.8)	4.7 (4.6)	2.2 (2.9)	2.6 (4.6)	1.2 (4.3)	3.2 (2.9)	3.0 (2.2)	2.5 (2.3)	17.3 (11.5)
Free sugars Mean (SD)	8.4 (8.7)	8.2 (6.2)	4.8 (8.8)	3.1 (4.1)	4.4 (4.4)	2.2 (2.9)	2.5 (4.4)	0.9 (3.1)	3.1 (2.8)	1.3 (1.5)	0.7 (1.7)	0.4 (0.7)
% TEI total sugars Mean (SD)	3.1 (3.1)	7.6 (3.3)	1.8 (3.6)	1.2 (1.6)	1.6 (1.4)	0.8 (1.2)	0.9 (1.4)	0.4 (1.3)	1.2 (1.1)	1.1 (0.8)	0.9 (0.8)	6.3 (4.0)
%TEI free sugars Mean (SD)	3.0 (3.1)	2.8 (1.9)	1.8 (3.6)	1.1 (1.4)	1.5 (1.3)	0.8 (1.2)	0.9 (1.3)	0.3 (0.9)	1.1 (1.0)	0.5 (0.5)	0.2 (0.6)	0.1 (0.3)
Freq. Mean (SD)	0.7 (0.6)	3.2 (1.3)	0.9 (0.9)	0.3 (0.3)	0.8 (0.8)	0.2 (0.3)	0.4 (0.5)	0.1 (0.3)	0.9 (0.5)	5.8 (1.7)	1.8 (0.7)	3.2 (1.7)
Prob. as snack	27.0	26.0	30.0	73.0	69.0	66.0	18.0	37.0	5.0	21.0	19.0	33.0
Prob. as meal	73.0	74.0	70.0	27.0	31.0	34.0	82.0	63.0	95.0	79.0	81.0	67.0

*TEI* total energy intake, *Freq* frequency, *RTEBC* ready-to-eat-breakfast-cereals

## Discussion

The main aim of this paper was to estimate TS and FS intake for 3-year-old preschoolers, identify the key food sources and to discuss the health, policy and research implications in the context of the recent WHO guidelines [37]. The results suggest that while there is a degree of subjectivity in assigning FS estimates, the overall figures were consistent with a previous analysis which used the same cohort data [10]. The TS and FS intakes contributed to 26.9 and 14.1% of TEI, respectively. Only a small minority (less than 4%) of children achieved the WHO conditional recommendation for the lower threshold for FS intake while three-quarters of children exceeded the higher maximum threshold of 10% TEI. ‘Fruit juices and smoothies’, ‘dairy products’ (including yoghurts and fromage frais), ‘soft drinks’ (including squashes, cordials and fruit juice drinks), ‘confectionary’ (chocolate and non-chocolate) and ‘cakes and biscuits’ were the key food sources for FS, contributing to more than three-quarters of total FS intake. For consumers only, ‘fruit juice and smoothies’, ‘dairy products’ and ‘soft drinks’ (including squashes, cordials and fruit juice drinks) contributed two-thirds of the total FS intake. The key sources of TS were ‘dairy products’, ‘fruit and vegetables’ and ‘fruit juice and smoothies’ contributing 63% of TS intake. Using these food groups and treating all snacks and main meals independently, the probability of consuming ‘chocolate’ and ‘non-chocolate confectionary’ and ‘cakes and biscuits’ as a snack ranged from 66 to 73% whereas the probability of consuming ‘fruit juice and smoothies’ or ‘non-diet soft drinks’ (including squashes, cordials and fruit juice drinks) as a snack was 27% or 30%, respectively.

Previous results reported that older Irish children (5–12 years old) had a frequency of intake of TS of four times per day which corresponded to a mean added sugar intake of 14.6% of TEI [38]. Our results suggested that younger children (3 years old) had a median frequency of five times per day of TS and four times per day of FS. The current advice to reduce both the amount of sugar and aim towards a maximum frequency of 1/day of sugary foods and drinks is aimed at meeting the WHO guidelines [4, 6, 25]. Intrinsic sugars which are part of the intact cellular structure of fruit or vegetables or lactose present in milk are generally not associated with negative health effects. Evidence supporting the updated WHO guidelines were primarily based on a systematic review of the relationship between sugar intake and dental caries [39] and between sugar and obesity [40]. Thus, the current recommendations focus on FS which are the main contributors to the risk of dental caries, obesity and other non-communicable diseases [6]. Our results were similar to those previously reported where a large proportion of children greatly exceed the recommended 10% FS as energy intake [12–15, 41, 42]. Most of the data available in the EU has reported on AS intake, rather than FS. Given the difference between AS and FS this suggests that the level of FS intake is probably higher. For example, AS intake in the USA in 2–5-year old is 13.4% of TEI, however, as noted in a recent commentary [9], these figures excluded FS in 100% natural fruit juice. In this analysis, the mean FS intake was 40 g/day, similar to that reported for AS intake for 4-year old children in the UK and Denmark [42]. Furthermore, as dietary surveys tend to under-estimate sugar intake, FS consumption is, probably, under-reported [10, 43].

Targeting the key food and beverage sources that are the largest contributors (for consumers) to FS intake would appear to be a sensible approach. However, it is important to know what the consumption distributions are, whether these targeted foods or beverages contribute to nutrient intake and whether their negative physiological effects are modified by other food components in the overall diet [24, 44]. Thus, strategies for reducing FS consumption for dental caries prevention may be different to those for targeting weight control and obesity risk. Providing dietary advice to patients regarding the reduction of ‘sugary snacks’ and limiting consumption to main mealtimes only, has long been a recommendation for the prevention of dental caries [23, 24]. As indicated by the high probability of consuming ‘chocolate’ and ‘non-chocolate confectionary’ and ‘cakes and biscuits’ as ‘snacks’ in this analysis, these foods could be more easily avoided when trying to target the frequency of intake and restrict consumption [23, 25, 45]. Currently, it would appear that children’s snack choices are dominated by foods high in FS [46] so substituting these snacks high in FS with healthier alternatives would seem one obvious dietary strategy to help reduce FS intake.

As a prerequisite for setting guidelines, targeting public health policy and measuring adherence to recommendations it is necessary to quantify the current intake of TS/FS/AS and the main food sources [8, 10, 12–15, 41]. It has been estimated that approximately three-quarters of all packaged foods and beverages sold in the USA contain AS [46]. Currently, food manufacturers in the EU are not required to include FS content in their labelling but under new regulations in the USA it will become a requirement to include AS on food labels from 2020. However, analytically it is not possible to distinguish TS and FS or AS [8, 32, 47] and most methods employed to estimate FS/AS have a degree of subjectivity and variation between country and product [13, 14, 41]. The problem is compounded by the lack of standardisation of terminology and methods added to the well-known difficulties that already exist in measuring food intake in young children [48]. While the leading sources of FS or AS intake tend to be the low nutrient, discretionary foods [12, 15, 41, 49] our analysis suggested that some nutrient-rich foods, such as sweetened yoghurts (dairy products), are also significant contributors of FS at this young age. Furthermore, even within the EU there are large variations between countries in the sugar content of some of these energy-dense, discretionary foods [42]. For example, RTEBC has been highlighted as a key source of AS/FS intake with efforts being made to reformulate these products as part of reducing overall FS consumption [42, 50, 51]. However, there are wide variations within and across countries in the sugar content of RTEBC and in our analysis these products contributed less than 8% of total FS intake. Thus, to reduce FS intake it is important to consider the most efficient products for reformulation given variations in content between countries and in consumption patterns at different ages [42, 50, 51].

Most analyses of cross-sectional data have reported an inverse association between the overall level of FS/AS intake and nutrient density [14, 38]. A number of researchers have suggested that food-based guidelines should focus on discouraging energy dense, nutrient-poor, discretionary food sources such as confectionary and soft drinks as a more practical approach to gaining a better overall nutrient intake than restricting all foods containing sugar [8, 12, 14]. This seems to be a sensible approach, particularly for age ranges where other key food sources such as RTEBC and sweetened dairy products still contribute significantly to overall nutrient intake. A recent systematic review investigating the association between consumption of food and drink containing FS close to bedtime and the risk of dental caries in children concluded that while there was a positive association, the quality of the evidence was very low [52]. However, the evidence supporting current recommendations to restrict bedtime consumption of food and drink containing FS was more consistent for preschool children than for older groups.

The SFQ in GUI did not capture approximately one-third of FS and one-quarter of TS intake suggesting that relying on modified short food questionnaires can result in significant underestimation of typical intake of food sources of sugar. This emphasised the importance of ensuring the most appropriate instrument is selected at survey design stage to achieve the optimal results within the constraints of resources. If, for example, body weight and height are the only physical parameters measured in a survey, allowing estimation of BMI and obesity, then a dietary intake instrument that can sufficiently capture total energy and habitual food intake would be appropriate. Looking at possible relationships between sugar intake and obesity without capturing one-third of FS consumption may lead to misleading conclusions. The most frequently consumed food and beverage that were *non*-*covered* included ‘RTEBC’, ‘fruit juices’ and ‘sugars and syrups’. As highlighted by our analysis and previous researchers ‘RTEBC’ and ‘fruit juices’ are items of consumption that have become difficult to classify as ‘healthy’ or ‘unhealthy’ as they can be an important source of nutrients for young children but also contain relatively high levels of FS [12, 23, 24, 53, 54].

Apart from the limitations of measuring dietary intake [55] there are other issues with terminology when researchers are trying to compare the frequency of intake of TS/FS including the definition of eating occasions or snacks [45, 56]. The lack of standardisation of commonly used terms plus the known phenomenon of under-reporting of snacks, particularly by subjects who are obese or overweight, will influence the number of eating occasions recorded and reported [56, 57]. There are known limitations with the methods used to assess dietary intake [55] in very young children [48]. Individuals also tend to reduce their actual consumption when intake is monitored, although for parent-reported data this may not be as problematic. However, parental recall of food intake is likely to lead to under-reporting [48] and this may be more pronounced with foods considered to be unhealthy. Under-reporters were not excluded from this analysis. As suggested in a recent review of TS and AS intakes in Europe [15] there is an urgent need to develop a standardised systematic methodology [32] to minimise the reporting of inappropriate estimates of AS or FS. However, our analysis was based on nationally representative data and used food intake estimates measured using a 4-day weighed food diary with a high level of researcher-participant interaction (3 visits over 4 days).

Public health interventions to encourage a reduction in the frequency of consumption and amount of foods high in FS/AS is to be welcomed [37, 50, 51]. Ultimately, what is required is a more precise understanding of the pattern of dietary sugar intake and the potential influence of FS/AS with other foods as part of a main meal and as snacks, on diet-related diseases such as obesity and dental caries.

## Conclusion

Accurate and reliable data on FS intake at the preschool age is a limiting factor in understanding consumption levels and key food sources. This analysis highlights the lack of standardised methods for FS estimation and the importance of using appropriate methods for quantifying sugar intake at the food level. Brief SFQ’s are not suitable techniques for understanding habitual sugar intake or comparing frequency of sugar intake patterns and will lead to bias when examining possible associations with disease. A large majority of 3-year-old Irish children do not meet the WHO recommended guidelines for FS intake and almost none meet the desired conditional recommendation. Consequently, it can be proposed that FS intake is excessively high, even at this early age, and reducing the intake of low nutrient, discretionary food and drink seems a reasonably pragmatic approach to achieving an overall reduction in FS consumption.

## References

[1] Allison DB, Bassaganya-Riera J, Burlingame B, Brown AW, le Coutre J, Dickson SL, van Eden W, Garssen J, Hontecillas R, Khoo CS, Knorr D, Kussmann M, Magistretti PJ, Mehta T, Meule A, Rychlik M, Vogele C (2015). Goals in Nutrition Science 2015-2020. Front in Nutr.

[2] SACN (2015) Carbohydrates and health report: SACN (Scientific advisory committee on nutrition). TSO. https://www.gov.uk/government/publications/sacn-carbohydrates-and-health-report. Accessed 3 June 2017

[3] Pyne V, Macdonald IA (2016). Update on carbohydrates and health: the relevance of the Scientific Advisory Committee on Nutrition report for children. Arch Dis Child.

[4] Moynihan P (2016). Sugars and dental caries: evidence for Setting a Recommended Threshold for Intake. Adv Nutr.

[5] Sheiham A, James WP (2015). Diet and dental caries: the pivotal role of free sugars reemphasized. J Dent Res.

[6] World Health Organization (2015). Guideline: sugars intake for adults and children.

[7] Marshall TA (2015). Nomenclature, characteristics, and dietary intakes of sugars. J Am Dent Assoc.

[8] Erickson J, Slavin J (2015). Total, added, and free sugars: are restrictive guidelines science-based or achievable?. Nutrients.

[9] Moynihan P, Makino Y, Petersen PE, Ogawa H (2018). Implications of WHO Guideline on Sugars for dental health professionals. Community Dent Oral Epidemiol.

[10] Newens KJ, Walton J (2016). A review of sugar consumption from nationally representative dietary surveys across the world. J Hum Nutr Diet.

[11] Brisbois TD, Marsden SL, Anderson GH, Sievenpiper JL (2014). Estimated intakes and sources of total and added sugars in the Canadian diet. Nutrients.

[12] Lei L, Rangan A, Flood VM, Louie JC (2016). Dietary intake and food sources of added sugar in the Australian population. Br J Nutr.

[13] Ruiz E, Rodriguez P, Valero T, Ávila J, Aranceta-Bartrina J, Gil Á, González-Gross M, Ortega R, Serra-Majem L, Varela-Moreiras G (2017). Dietary intake of individual (free and intrinsic) sugars and food sources in the Spanish population: findings from the ANIBES study. Nutrients.

[14] Gibson S, Francis L, Newens K, Livingstone B (2016). Associations between free sugars and nutrient intakes among children and adolescents in the UK. Br J Nutr.

[15] Azais-Braesco V, Sluik D, Maillot M, Kok F, Moreno LA (2017). A review of total & added sugar intakes and dietary sources in Europe. Nutr J.

[16] Farajian P, Risvas G, Panagiotakos DB, Zampelas A (2016). Food sources of free sugars in children’s diet and identification of lifestyle patterns associated with free sugars intake: the GRECO (Greek Childhood Obesity) study. Public Health Nutr.

[17] Bailey RL, Fulgoni VL, Cowan AE, Gaine PC (2018). Sources of added sugars in young children, adolescents, and adults with low and high intakes of added sugars. Nutrients.

[18] Mis NF, Braegger C, Bronsky J, Campoy C, Domellöf M, Embleton ND, Hojsak I, Hulst J, Indrio F, Lapillonne A (2017). Sugar in infants, children and adolescents: a position paper of the European society for paediatric gastroenterology, hepatology and nutrition committee on nutrition. J Pediatr Gastroenterol Nutr.

[19] King JM (1978). Patterns of sugar consumption in early infancy. Commun Dent Oral Epidemiol.

[20] Rossow I, Kjaernes U, Holst D (1990). Patterns of sugar consumption in early childhood. Commun Dent Oral Epidemiol.

[21] Friel S, Nolan G, Kelleher C (1996). Changes in the food chain since the time of the Great Irish Famine.

[22] Chi DL, Luu M, Chu F (2017). A scoping review of epidemiologic risk factors for pediatric obesity: implications for future childhood obesity and dental caries prevention research. J Public Health Dent.

[23] Moynihan PJ (2002). Dietary advice in dental practice. Br Dent J.

[24] Moynihan P, Petersen PE (2004). Diet, nutrition and the prevention of dental diseases. Public health Nutr.

[25] van Loveren C (2018). Sugar restriction for caries prevention: amount and frequency. Which is more important?. Caries Res.

[26] Crowe M, O’Sullivan M, McNulty BA, Cassetti O, O’Sullivan A (2018). Data mapping from food diaries to augment the amount and frequency of foods measured using short food questionnaires. Front Nutr.

[27] Murray A, Quail A, McCrory C, Williams J (2013). A summary guide to wave 2 of the infant cohort (at 3 years) of Growing Up in Ireland.

[28] Quail A, Williams J, McCrory C, Murray A, Thornton M (2011). Sample design and response in wave 1 of the infant cohort (at 9 months) of Growing Up in Ireland.

[29] Walton J, Flynn A (2013). Nutritional adequacy of diets containing growing up milks or unfortified cow’s milk in Irish children (aged 12–24 months). Food Nutr Res.

[30] Universities Nutrition Alliance (2012) National Preschool Nutrition Survey 2010-11. http://www.iuna.net/?p=169. Accessed 15 May 2017

[31] Crowe MJ (2018) Diet and other risk indicators associated with dental problems in Irish preschool children. Dissertation, Trinity College Dublin. http://hdl.handle.net/2262/83838

[32] Louie JC, Moshtaghian H, Boylan S, Flood VM, Rangan AM, Barclay AW, Brand-Miller JC, Gill TP (2015). A systematic methodology to estimate added sugar content of foods. Eur J Clin Nutr.

[33] Team R (2015) RStudio: integrated development for R. RStudio, Inc, Boston, MA. http://www.rstudio.com/42. Accessed 12 Jan 2018

[34] World Health Organization (2003). Diet, nutrition and the prevention of chronic diseases. WHO technical report series.

[35] Schofield W (1985). Predicting basal metabolic rate, new standards and review of previous work. Hum Nutr Clin Nutr.

[36] Torun B (1996). Energy requirements and dietary energy recommendations for children and adolescents 1 to 18 years old. Eur J Clin Nutr.

[37] Breda J, Jewell J, Keller A (2018). The importance of the world health organization sugar guidelines for dental health and obesity prevention. Caries Res.

[38] Joyce T, McCarthy SN, Gibney MJ (2008). Relationship between energy from added sugars and frequency of added sugars intake in Irish children, teenagers and adults. Br J Nutr.

[39] Moynihan PJ, Kelly SAM (2014). Effect on caries of restricting sugars intake: systematic review to inform WHO guidelines. J Dent Res.

[40] Te Morenga L, Mallard S, Mann J (2013). Dietary sugars and body weight: systematic review and meta-analyses of randomised controlled trials and cohort studies. BMJ.

[41] Sluik D, van Lee L, Engelen AI, Feskens EJ (2016). Total, free, and added sugar consumption and adherence to guidelines: the Dutch national food consumption survey 2007–2010. Nutrients.

[42] World Health Organization (2017) Incentives and disincentives for reducing sugar in manufactured foods. An exploratory supply chain analysis. A set of insights for member states in the context of the WHO European food and nutrition action plan 2015–2020. WHO, Copenhagen, Denmark. http://www.euro.who.int/en/health-topics/disease-prevention/nutrition/publications/2017/incentives-and-disincentives-for-reducing-sugar-in-manufactured-foods-2017. Accessed 20 Feb 2018

[43] Livingstone MB, Black AE (2003). Markers of the validity of reported energy intake. J Nutr.

[44] Johansson I, Holgerson PL, Kressin NR, Nunn ME, Tanner AC (2010). Snacking habits and caries in young children. Caries Res.

[45] Marshall TA, Broffitt B, Eichenberger-Gilmore J, Warren JJ, Cunningham MA, Levy SM (2005). The roles of meal, snack, and daily total food and beverage exposures on caries experience in young children. J Public Health Dent.

[46] Popkin BM (2014). Sugar consumption in the food and beverage supply across the globe. Dietary sugars and health.

[47] Stephen A, Alles M, de Graaf C, Fleith M, Hadjilucas E, Isaacs E, Maffeis C, Zeinstra G, Matthys C, Gil A (2012). The role and requirements of digestible dietary carbohydrates in infants and toddlers. Eur J Clin Nutr.

[48] Magarey A, Watson J, Golley RK, Burrows T, Sutherland R, McNaughton SA, Denney-Wilson E, Campbell K, Collins C (2011). Assessing dietary intake in children and adolescents: considerations and recommendations for obesity research. Int J Pediatric Obes.

[49] Welsh JA, Figueroa J (2017). Intake of Added Sugars During the Early Toddler Period. Nutr Today.

[50] Public Health England (2017) Sugar reduction: achieving the 20%. A technical report outlining progress to date, guidelines for industry, 2015 baseline levels in key foods and next steps

[51] Public Health England (2015) Sugar reduction: the evidence for action. www.gov.uk/government/uploads/system/uploads/attachment_data/file/470179/Sugar_reduction_The_evidence_for_action.pdf. Accessed 12 Jan 2016

[52] Baghlaf K, Muirhead V, Moynihan P, Weston-Price S, Pine C (2018). Free sugars consumption around bedtime and dental caries in children: a systematic review. JDR Clin Transl Res.

[53] Priebe MG, McMonagle JR (2016). Effects of ready-to-eat-cereals on key nutritional and health outcomes: a systematic review. PLoS One.

[54] Dye BA, Shenkin JD, Ogden CL, Marshall TA, Levy SM, Kanellis MJ (2004). The relationship between healthful eating practices and dental caries in children aged 2–5 years in the United States, 1988–1994. J Am Dent Assoc.

[55] Thompson F, Subar A, Coulston A, Boushey C, Ferruzzi M (2013). Dietary assessment methodology. Nutrition in the prevention and treatment of disease.

[56] Leech RM, Worsley A, Timperio A, McNaughton SA (2015). Understanding meal patterns: definitions, methodology and impact on nutrient intake and diet quality. Nutr Res Rev.

[57] Gibney MJ, Wolever TMS (1997). Periodicity of eating and human health: present perspective and future directions. Br J Nutr.

